# Neonatal ICU antibiotic use trends within an integrated delivery network

**DOI:** 10.1186/s13756-022-01057-3

**Published:** 2022-01-31

**Authors:** Gregory Boverman, Christine Perez, Shruti Vij, Kristen Tgavalekos, Shreyas Ravindranath, Cornel Antonescu, Bobbi Chambers-Hawk

**Affiliations:** 1 Philips Research, Cambridge, MA 02141 USA; 2 Philips Clinical Professional Services, Cambridge, MA 02141 USA; 3 Banner Health, Phoenix, AZ 85006 USA

**Keywords:** Antibiotics, ASP, Neonates, NICU, AUR, IDN

## Abstract

**Background and objectives:**

There is a need for robust antibiotic stewardship programs (ASPs) in the neonatal population. This study's objectives were to assess neonatal antibiotic use practices over an extended period across an integrated delivery network (IDN), including six Neonatal Intensive Care Units (NICUs), to identify those most successful practices reducing use rates.

**Methods:**

A retrospective cohort study was conducted, including 15,015 NICU admissions from an integrated delivery network, across six hospitals over eight years (50% Level III and 50% Level II) computing antibiotic use rates (AURs) stratified by usage: in the first few days of the stay vs. later in the stay and by gestational age. Several metrics were examined for assumptions of strong correlation with AUR: (1) the percentage of infants given antibiotics early in their stays and (2) durations of courses of antibiotics.

**Results:**

Results conclude a wide variation in AURs and trends that these rates followed over time. However, there was a decrease in overall AUR from 15.7–16.6 to 10.1–10.8%, with four of the six NICUs recording statistically significant reductions in AUR vs. their first year of measurement. Specifically, the level III NICUs overall AUR decreases from 15.1–16.22 to 8.6–9.4%, and level II NICUs overall AUR 20.3–24.4 to 14.1–16.1%. A particularly successful level II NICU decreased its AUR from 22.9–30.6 to 5.9–9.4%.

**Conclusion:**

To our knowledge, this is the first study to utilize data analytics at an IDN level to identify trends in AUR, We have identified practices that allowed an institution to reduce NICU AURs significantly, and which, if done as a standard practice, could be replicated on a broader scale.

## Background

The early use of antibiotics in the neonatal period increases antimicrobial resistance [1]. In 2015, the World Health Organization (WHO) identified antimicrobial resistance as an enduring public health threat and published a Global Action Plan [2] and in the same year it was estimated that 214,000 neonatal sepsis deaths each are attributable to resistant pathogens [3]. The most commonly utilized medications in the Neonatal Intensive Care Unit (NICU) are antibiotics, with greater than 80% of all NICU admissions receiving antibiotics during their hospital course [4, 5].

Life-threatening infections in the NICU generally fall into the two broad categories of early-onset sepsis (EOS) and late-onset sepsis (LOS). A diagnosis of EOS occurs within the first 48–72 h of life with an incidence of 0.3–1.0 per 1000 live births, and a LOS diagnosis is after the initial 48–72 h with a higher rate of 2.2 per 1000 live births [5, 6].The underlying causative organisms for EOS and LOS vary, requiring different approaches to antibiotic stewardship.

### Adverse outcomes of early antibiotic use in neonates

Antibiotic use in the NICU in the first week of life increases morbidity and mortality, results in mother and child separation, and increasing healthcare costs [7, 8]. Early or prolonged empiric antibiotic use for preterm neonates results in an increased risk of necrotizing enterocolitis (NEC), infections, and mortality [5, 9, 10]. For instance, a recent retrospective study of very low birth weight neonates (less than 1500 g and 32 6/7 weeks’ gestation) demonstrates for each additional day of antibiotics; there is a 24% increase in the risk for the development of sepsis, NEC, or death [9]. Furthermore, utilization of antibiotics early and for a prolonged duration reduces the gut microbiota increasing antibiotic resistance and altering the immune system early in life [1, 11–13]. Alterations to microbes early in life increase the risk for autoimmune disorders, obesity, and allergic diseases greater than the two-fold risk for asthma [11, 13, 14].

### Antibiotic stewardship programs in the NICU

Consequently, there is a need for robust antibiotic stewardship programs (ASPs), particularly in the neonatal population, who have underdeveloped immune systems. ASPs are hospital-based programs dedicated to improving antibiotic use by optimizing the treatment of infections and reducing adverse events associated with antibiotics. The Infectious Disease Society of America and the Society for Healthcare Epidemiology of America recommend that APS, compromised of appropriate antibiotic selection, proper dosing, therapy duration, as well as the route of administration, be implemented in the NICU to decrease inappropriate use and resistance [15]. For instance, inappropriate and overuse of vancomycin results in high colonization rates leading to outbreaks of vancomycin-resistant enterococci (VRE) [11]. However, the implementation of a vancomycin guideline resulted in a significant decrease in vancomycin use in two tertiary NICUs of 35% and 65%, demonstrating the applicability of ASPs in the NICU [16]. Another retrospective chart review showed that implementing ASPs into the NICU could decrease total antibiotic days of therapy (DOT) by 18%, decrease targeted broad-spectrum antibiotic DOT by 70%, and decrease vancomycin use in two NICUs by 35% and 62% [17].

Implementing an ASP provides clinical management, surveillance interventions, and work strategies, including prescribing practices such as choosing the correct antibiotic, dosing, course duration, and monitoring, as well as educating healthcare workers [18]. Following the implementation of ASPs, a tertiary NICU in the United Kingdom significantly reduced overall antibiotic use by 43% from a median of 347 antibiotic use days to 198 antibiotic use days per 1000 patient days, reduced median days of antibiotic use at discharge from three to two days, and decreased practice variations [19]. Importantly, the use of an ASP in a level III-IV NICU resulted in a reduction from 543 to 380 DOT per 1000 patient days and decreased rates of late-onset sepsis from 11.4 to 6.5% without increasing readmission rates [20].

Despite the prevalence of ASPs, there continues to be significant variability in antibiotic use rate (AUR), defined as the number of patient days that neonates were exposed to antibiotics (1 or more) per 100 patient days. Alarmingly, misuse of antibiotics in the NICU may be as high as 20–50% [21]. Analysis of 127 NICUs in California revealed a 40-fold variance in AUR from 2.4 to 97.1% correlating with the NICU level of care [22]. Level III-IV NICUs (regional, community) had a 7 to 12-fold variance in AUR while the variation in AUR at level II (intermediate) NICUs was significantly higher at 31-fold [22].

The American Academy of Pediatrics (AAP) endorses the use of a multivariate risk calculator to guide initiation of antibiotics in infants > 34 weeks gestation at risk for early-onset sepsis with prompt discontinuation of antibiotics after 36–48 h if blood culture remains sterile [6]. A more extensive cohort study of 204,485 neonates at 35 or greater week’s gestation utilization of the EOS calculator reduced antibiotic administration within the first 24 h of birth from 5.0 to 2.6% [23]. Another meta-analysis review of 175,752 newborns applying the EOS calculator resulted in a lower relative risk for antibiotic therapy with no higher readmission rates [24].

Utilization of the NICU BacT/Alert microbial detection technology allows for detecting infection in as few as 24–36 h [25]. In fact, in one review of 845 neonatal blood cultures, all gram-negative isolates were identified by 26 h and many as early as nine hours after inoculation [25]. However, a study utilizing the National Antimicrobial Prescribing Survey Australian Database with 215 neonates from 39 different hospital audits revealed 22% of antibiotics were given beyond 48 h with 9% more than 72 h despite a confirmed infection in only 4.2% neonates [26].

The Center for Disease Control and Prevention (CDC) [27] developed seven standardized core elements of ASPs, including leadership, accountability, drug expertise, actions, tracking, reporting, and education. In a recent quality audit, none of the 143 participating NICUs report following all seven of these guidelines, while noting a median AUR of 17% [21]. Incorporating an automatic stop order for antibiotics at 48-h in the electronic order set on admissions is one strategy shown to significantly reduce antibiotic use by 30–38%, creating a mandatory review and opt-in approach for use beyond 48-h [28].

A collaboration of 146 NICUs participating in the Choosing Antibiotics Wisely Campaign, a QI collaborative by Vermont Oxford Network online program with interactive web sessions, decreased the median AUR from 16.7 to 12.1%, a 34% relative risk reduction [29]. Concurrently, participating NICUs increased use of the CDC seven ASP core measures of leadership 15.4–68.8%, accountability 54.5–95%, drug expertise 61.5–85.1%, actions 21.7–72.35%, tracking 14.7–78%, reporting 6.3–17.7% and education 32.9–87.2% highlighting the necessity for leadership engagement and new methods for reporting antibiotic practices[29].While many hospitals have detailed ASP protocols for adult patients, similar NICU protocols are not commonly implemented. Our study aimed to explore AUR changes in the NICUs of an IDN to expand primary factors driving change in antibiotic prescription practices in the NICU and provide a tool for reporting on ASPs.

## Methods

We collected retrospective data of all NICU admissions at birth (age 0 to 1 day) to a medium-sized integrated delivery network (IDN) from years 2010 to 2017, excluding NICU readmissions (which accounted for less than 6% of admissions). The description of the resulting cohort is shown in Table 1. As expected, Level III NICUs cared for neonates with lower gestational ages, higher mortality rates, and longer lengths of stay in comparison to Level II NICUs in the IDN.

**Table 1:** Cohort for retrospective study of antibiotic use

NICU	Number of admissions	Median LOS	Median Gestational Age	Percent Mortality	Median Weight (g)	NICU Beds	Level
Hospital 1	4633	15	34.45	3.4	2170	76	(III)-Specialized Services
Hospital 2	2969	15	35	2.8	2292.24	74	(III)-Specialized Services
Hospital 3	2583	9	36	3.4	2330	17	III
Hospital 4	2016	4	37.1	0	3030		(II)-Specialized Services
Hospital 5	1414	5	37	0.2	2602.5	20	(IIE)
Hospital 6	1400	4	37.85	0.2	2256.5	13	(II)

For each NICU and each year, all corresponding admissions were identified. For the AUR computation, the numerator is the sum of the number of days of antibiotics for all admitted neonates, and the denominator is the sum of the lengths-of-stay divided by 100 (i.e., the denominator is per 100 patient-days). Note that fractional patient days were rounded-up to the nearest whole number of days, an approach that was standardized across hospitals. Note also that days where multiple antibiotics were given were counted as single antibiotic days.

However, unlike in previous studies, the AUR was utilized as a rate parameter inferred from the discrete measurements. Furthermore, this is akin to determining if a coin is fair from a limited number of trials—our confidence in the estimate of the rate parameter increases as the number of trials increases. Notably, confidence bounds can be quite wide; however, it is plausible to infer statistical statements about improvements over time. For each hospital and each year after the first, the statistical significance (i.e., p-value) was calculated for the one-sided z-proportions test for the reduction in the proportion of antibiotic days as a fraction of patient days, as compared to both the previous year and the first year of recorded data for that institution. Additionally, NICUs joined the IDN at different points in time, somewhat complicating the analysis.

Interestingly, particularly for level II NICUs, nearly all antibiotics prescribed were given in the first three days of life. For example, for Hospital 4, between 90.3 and 98.9% of all antibiotic days belonged to courses starting in the first three days of life, presumably for empiric treatment of infections believed to be early-onset sepsis (EOS). Given recent research showing that the administration of antibiotics to only those neonates with a demonstrable risk of sepsis hypothesized that NICUs could significantly reduce their antibiotic usage by lowering the use of early antibiotics. Statistical bounds computation was utilized for the proportions of neonates given early antibiotics, using the z-proportions test to assess differences in rates over time.

We also considered durations of antibiotics, where a “course” of antibiotics was defined as a sequence of doses where each dose was separated by less than 24 h. We considered courses of antibiotics associated with bacterial blood cultures (taken up to two days before the start of the course until the end of the course) as other uses of antibiotics may not be explicitly for suspicion of inspection but for other purposes (e.g., preparation for surgery). Bootstrap sampling was performed to compute statistical bounds for the mean duration of antibiotics. Utilization of the non-parametric Mann–Whitney U-test to assess whether there were statistically significant differences in mean course durations both year-to-year and compared to the first year for which data were available a given NICU. Finally, although these results are reported elsewhere, an assessment was completed for the positive and negative predictive values of provisional bacterial culture results at 24, 36, and 48 h.

## Results

**Fig. 1:**
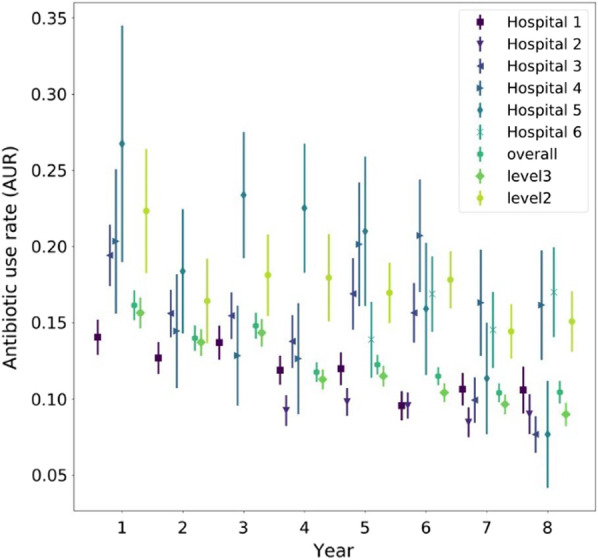
Visualization of AUR improvements over time

The AUR for the six NICUs as a function of time are shown in Table 2. Indication with a “ + ” statistically significant changes vs. the first year for which data were available and with a “*” year-over-year reductions in AUR. As mentioned previously, the overall AUR decreased substantially, and all but two NICUs achieved significant reductions in antibiotic use vs. the first year. These two NICUs (numbers 2 and 6) had limited data compared to the others. The AURs are visualized in Fig. 1, which shows the overall decreasing trend and shows that the confidence bounds for level III and level II NICU AURs are generally non-overlapping, meaning that it is perhaps difficult to compare their usages of antibiotics directly. For comparable NICUs within the same IDN, there was significant variability in improvements, with NICU 3 and 5 reducing their usage significantly, while NICUs 1 and 4 achieved more modest reductions, and NICU 6 appears to have increased its usage over time.

**Table 2:** Antibiotic use rate for NICUs as a function of time

NICU	Year 1	Year 2	Year 3	Year 4	Year 5	Year 6	Year 7	Year 8
Hospital 1	13.5–14.6%	12.2–13.2%* +	13.1–14.3%	11.4–12.4%* +	11.4–12.5% +	9.1–10.0%* +	10.1–11.2% +	9.8–11.4% +
Hospital 2				8.7–9.8%	9.4–10.3%	9.1–10.0%	8.0–9.0%* +	8.4–9.7%
Hospital 3	18.4–20.4%	14.8–16.4%* +	14.7–16.2% +	12.9–14.6%* +	15.7–18.1% +	14.7–16.6% +	9.2–10.7%* +	7.1–8.3%* +
Hospital 4	18.0–22.7%	12.6–16.3%* +	11.2–14.5% +	10.8–14.5% +	18.1–22.2%	18.9–22.6%	14.6–18.1%* +	14.4–18.0% +
Hospital 5	22.9–30.6%	16.3–20.4%* +	21.3–25.5%	20.4–24.6% +	18.5–23.5% +	13.8–18.1%* +	9.5–13.2%* +	5.9–9.4%* +
Hospital 6					12.6–15.1%	15.6–18.1%	13.3–15.8%*	15.5–18.5%
Overall	15.7–16.6%	13.6–14.4%* +	14.4–15.2% +	11.4–12.1%* +	11.9–12.6% +	11.2–11.8%* +	10.1–10.7%* +	10.1–10.8% +
Level 3	15.1–16.2%	13.3–14.2%* +	13.9–14.8% +	11.0–11.6%* +	11.2–11.8% +	10.1–10.7%* +	9.3–10.0%* +	8.6–9.4%* +
Leve l 2	20.3–24.4%	15.0–17.8%* +	16.8–19.5% +	16.5–19.4% +	16.0–18.0% +	16.9–18.8% +	13.5–15.3%* +	14.1–16.1% +

The proportion of neonates given antibiotics starting in the first three days of life is shown in Table 3. Here the improvement was consistent, and all NICUs saw statistically significant improvements, with varying levels. Notably, the improvements were relatively recent, with level III NICUs seeing improvement first, in year 5. In contrast, some of the level II NICUs, such as NICU 4, only saw a sustained improvement in year 8. However, hospital 5 decreased the percentage of neonates receiving early antibiotics by approximately 50%, likely accounting for a large proportion of the overall AUR reduction, given that for level II NICUs almost all antibiotics are early antibiotics (as evidenced by the relatively shorter lengths of stay).

**Table 3:** Ranges for percentages of neonates receiving early antibiotics as a function of time

NICU	Year 1	Year 2	Year 3	Year 4	Year 5	Year 6	Year 7	Year 8
Hospital 1	62.9–70.3%	63.3–70.9%	63.0–70.7%	60.6–68.1%	63.2–70.9%	56.7–64.7%* +	53.9–63.0% +	49.7–61.3% +
Hospital 2				57.3–65.9%	52.2–59.8%* +	51.9–59.5% +	50.4–59.5% +	50.0–60.5% +
Hospital 3	67.2–76.7%	59.2–68.6%* +	62.1–71.6%	59.0–69.7% +	55.9–68.7% +	53.0–65.0% +	54.7–66.6% +	44.0–54.5%* +
Hospital 4	44.4–62.5%	37.0–51.0%	28.4–41.0%* +	34.4–48.5% +	43.0–57.0%	59.6–72.4%	46.6–59.6%*	35.3–48.9%* +
Hospital 5	47.8–75.6%	47.4–65.0%	56.9–71.1%	52.3–66.8%	45.7–62.0%	40.0–56.5%	25.8–40.1%* +	16.8–32.7% +
Hospital 6					54.9–67.6%	51.2–61.6%	41.6–52.0%* +	44.0–54.8% +
Overall	63.9–69.4%	59.0–64.3%* +	58.6–63.8% +	58.5–63.0% +	57.5–61.8% +	56.0–60.1% +	50.7–55.1%* +	46.7–51.5%* +
Level 3	65.6–71.4%	62.9–68.8%	63.8–69.8%	60.9–65.9%* +	58.8–63.8% +	55.7–60.7%* +	54.7–60.4% +	50.0–56.3%* +
Level 2	48.2–63.5%	43.2–54.3%	42.8–52.6% +	45.2–55.4%	51.4–59.5%	54.0–61.3%	42.0–49.2%* +	39.0–46.6% +

Quantitatively speaking, there are two main contributors to the AUR: firstly, the proportion of neonates receiving antibiotics and, secondly, the duration of antibiotics they were given. After looking into the first question, we turned our attention to antibiotic durations, generating the results shown in Table 4. Here, there was a decidedly more mixed performance, with only three of six NICUs seeing statistically significant improvements vs. the first year when data were available for each NICU. Interestingly, there were reversal cases, such as NICU 4, which appears to have experienced an increase in mean duration in year eight. While mean durations of antibiotic courses have overall decreased considerably, there are still many cases well above two days.

**Table 4:** Mean durations of courses of antibiotics associated with cultures as a function of time (units of days)

NICU	Year 1	Year 2	Year 3	Year 4	Year 5	Year 6	Year 7	Year 8
Hospital 1	3.1–3.5	3.0–3.5	3.1–3.7	2.6–3.0* +	2.5–2.9 +	2.2–2.7* +	2.5–3.1 +	2.0–2.6* +
Hospital 2				2.2–2.6	2.4–2.9	2.6–3.1	2.3–2.8	2.2–2.9
Hospital 3	3.2–3.8	3.4–3.9	2.9–3.6* +	2.5–3.0* +	2.4–3.0 +	2.6–3.5 +	2.3–2.9 +	1.9–2.4 +
Hospital 4	2.6–3.7	1.7–2.1* +	1.8–2.4 +	1.5–1.6 +	1.9–2.3 +	2.0–2.5 +	1.7–2.0* +	2.3–3.0
Hospital 5	2.8–4.9	2.6–3.4	2.7–3.4	2.4–3.1	2.3–2.9	1.8–2.4* +	1.6–2.4 +	1.5–2.7 +
Hospital 6					1.9–2.2	2.2–2.7	1.9–2.3	1.9–2.2
Overall	3.2–3.5	3.1–3.4	3.0–3.4* +	2.5–2.7* +	2.5–2.7 +	2.5–2.7* +	2.3–2.6 +	2.2–2.5 +
Level 3	3.2–3.6	3.2–3.6	3.1–3.6* +	2.5–2.8* +	2.5–2.8 +	2.6–2.9 +	2.5–2.8 +	2.2–2.5 +
Level 2	2.8–3.9	2.2–2.6* +	2.4–2.9 +	2.0–2.5* +	2.1–2.3 +	2.1–2.5 +	1.9–2.1* +	2.1–2.4 +

Findings show strong evidence that in many cases, courses of antibiotics with a negative provisional result can be stopped as early as after 36 h and, in most cases, by 48 h. In analyzing the results of blood cultures overall for all six institutions, we found that the negative predictive values (specificities) of negative culture results at 24, 36, and 48 h were 96.9%, 98.9%, and 99.5%, respectively. The sensitivities of positive culture results at the same time intervals were 81.7%, 93.9%, and 97.3%. Discontinuing a course of antibiotics at 24 h risks missing approximately 18% of positive cases, but this risk drops to approximately 6% at 36 h and less than 3% at 48 h.

## Discussion

The substantial sample size of 15,015 NICU admissions from a large IDN with the inclusion of both Level II and III NICU's over eight years provides insights into antibiotic stewardship. A data-driven approach provides an additional tool for assessing NICU ASPs, including AUR, reporting, and tracking ASPs and identifying best practices for reducing unnecessary early antibiotic use in the neonatal period. From 2010 to 2017, overall AUR declined from 15.7–16.6 to 10.1–10.8%. Furthermore, the utilization of data analytics at the IDN level over eight years highlights the improvement in ASPs while identifying areas for improvements and best practices. In the study, a variance was seen in antibiotic practices in the six NICUs, noting one of the Level II NICUs significantly reduced AUR over the study's course despite previous studies indicating level II's have higher rates of AUR. Also, coinciding with other findings, the timing of negative predictive value of blood cultures was 98.9% at 36 h and 99.5% 48 h, providing further justification for ASPs practices such as a 48-h electronic, hard stop admission order. Two successful practices were identified, including a reduction in the proportion of late preterm (> 34 weeks gestational age) neonates receiving empiric antibiotics by a factor of three and a decrease in the duration of empiric antibiotic therapy to approximately 1.5 days for all gestational ages.

The WHO stresses the importance of reducing unnecessary antibiotic use to decrease antimicrobial resistance, which has an estimated cost to society of 1.2 trillion dollars in health expenditures by 2050 [30]. Lowering rates of AUR and early antibiotic use with a robust ASP in the NICU will aid in decreasing the burden of antimicrobial resistance and adverse outcomes through the neonatal period and beyond. Future studies are needed for predictive modeling of LOS and qualitative analysis of best practices for ASPs.

Although antimicrobial resistance was deemed a potential problem during the period in question, and there was some institutional understanding of the need to limit antibiotics in the early postnatal period, few IDN-wide practice standards in place codified best practices. This situation has recently changed, introducing an automatic 48-h stop for antibiotics orders and introducing a new element in the electronic medical record across all of the studied hospitals, computing and reporting the Kaiser Permanente risk score for early-onset sepsis mainly based on maternal risk factors. Incorporating automatic 48-h stops for antibiotic orders has been shown to reduce antibiotic use by 25–35% [28]. While a review of 175,752 newborns across 13 studies revealed utilization of an early onset calculator in the NICU significantly reduces empirical antibiotic therapy, [24] noting in a study of 204,485 newborns 35 weeks gestation or later utilization of the EOS reduced blood cultures by 9.6% with a 2.4% reduction in empiric antibiotic use in first 24 h [23]. The most significant AUR reduction during our study period was hospital five, who also participated in a statewide perinatal collaborative to improve antibiotic stewardship in the NICU. Coinciding, in a national NICU collaborative for Choosing Antibiotics Wisely, participants increased all seven domains of the CDC core antibiotic stewardship elements [29]. Reducing AUR in NICU and adhering to CDC antibiotic stewardship's core elements requires a multidisciplinary collaboration at the national, state, and organizational levels while incorporating unit-specific practice changes.

Therefore, potentially the variances observed in our study have been reduced with the introduction of these IDN-wide standards, which may be a future study topic. Despite these recent changes, we feel that our study is of current relevance, as many institutions may not have put into place system-wide or hospital-wide neonatal antibiotic use standards, and maybe informed to find variances in their use of antibiotics in the neonatal ICU setting, using techniques similar to those described here.

Our study's main limitation was that it was an observational retrospective cohort in a single IDN in one part of the country, which may not be reflective of the entire population. The data of the study is quantitative, limiting knowledge on actual protocols and ASPs per NICU. Another limitation, pointed-out to us by one of the anonymous reviewers, is that NICUs do often treat “older” neonates and that there is significant usage of antibiotics for peri-operative purposes, for example after surgery to treat congenital heart defects [31]. This aspect of antibiotic usage may be the subject of a future study.

## Conclusion

We conducted a retrospective cohort study of NICU antibiotic use over an extended period within a multi-hospital IDN, overall showing a reduction in AUR from 15.7–16.6 to 10.1–10.8% over eight years. Despite the overall reduction, there was wide variation in the rates of decrease in AURs over time, even within NICU classes. We identified specific practices, namely reducing antibiotic use rates due to suspicion of early-onset sepsis and reducing the duration of antibiotics for culture-negative cases, that appeared to contribute most significantly to the overall reductions over time for the most successful NICUs.

## Data Availability

The data that support the findings of this study are available from Banner Health but restrictions apply to the availability of these data, which were used under license for the current study, and so are not publicly available. Data are however available from the authors upon reasonable request and with permission of Banner Health.

## Abbreviations

ASP: Antibiotic stewardship program
NICU: Neonatal Intensive Care Unit
IDN: Integrated delivery network
AUR: Antibiotic use rate
NEC: Necrotizing enterocolitis
EOS: Early-onset sepsis
CDC: Centers for disease control (USA)
IRB: Institutional review board
